# New birth weight reference standards customised to birth order and sex of babies from South India

**DOI:** 10.1186/1471-2393-13-38

**Published:** 2013-02-14

**Authors:** Velusamy Saravana Kumar, Lakshmanan Jeyaseelan, Tunny Sebastian, Annie Regi, Jiji Mathew, Ruby Jose

**Affiliations:** 1 Department of Biostatistics, Christian Medical College and Hospital, Vellore, 632004, India; 2 Department of Obstetrics and Gynaecology, Christian Medical College and Hospital, Vellore, 632004, India

**Keywords:** Reference, Foetal growth, Birth weight, Gestational age, Preterm, Modelling, Box–Cox t, Cubic spline smoothing

## Abstract

**Background:**

The foetal growth standards for Indian children which are available today suffer due to methodological problems. These are, for example, not adhering to the WHO recommendation to base gestational age on the number of completed weeks and secondly, not excluding mothers with risk factors. This study has addressed both the above issues and in addition provides birthweight reference ranges with regard to sex of the baby and maternal parity.

**Methods:**

Data from the labour room register from 1996 to 2010 was obtained. A rotational sampling scheme was used i.e. the 12 months of the year were divided into 4 quadrants. All deliveries in January were considered to represent the first quadrant. Similarly all deliveries in April, July and October were considered to represent 2^nd^, 3^rd^ and 4^th^ quadrants. In each successive year different months were included in each quadrant. Only those mothers aged 20–39 years and delivered between 24 to 42 weeks gestational age were considered. Those mothers with obstetric risk factors were excluded. The reference standards were fitted using the Generalized Additive Models for Location Scale and Shape (GAMLSS) method for Box – Cox t distribution with cubic spline smoothing.

**Results:**

There were 41,055 deliveries considered. When women with risk factors were excluded 19,501 deliveries could be included in the final analysis. The male babies of term firstborn were found to be 45 g heavier than female babies. The mean birthweights were 2934 g and 2889.5 g respectively. Similarly, among the preterm babies, the first born male babies weighed 152 g more than the female babies. The mean birthweights were 1996 g and 1844 g respectively.

In the case of later born babies, the term male babies weighed 116grams more than the females. The mean birth weights were 3085 grams and 2969 grams respectively. When considering later born preterm babies, the males outweighed the female babies by 111 grams. The mean birthweights were 2089 grams and 1978 grams respectively. There was a substantial agreement range from k=.883, (p<.01) to k=.943, (p<.01) between adjusted and unadjusted percentile classification for the subgroups of male and female babies and first born and later born ones.

Birth weight charts were adjusted for maternal height using regression methods. The birth weight charts for the first born and later born babies were regrouped into 4 categories, including male and female sexes of the babies. Reference ranges were acquired both for term and preterm babies.

With economic reforms, one expects improvement in birthweights. The mean (sd) birthweights of the year 1996 was 2846 (562) as compared to year 2010 (15 years later) which was 2907 (571). There was only a difference of 61 grams in the mean birthweights over one and a half decade.

**Conclusion:**

New standards are presented from a large number of deliveries over 15 years, customised to the maternal height, from a south Indian tertiary hospital. Reference ranges are made available separately for first born or later born babies, for male and female sexes and for term and preterm babies.

## Background

Investigators and researchers have used the reference standards which were developed globally due to lack of availability of population or country specific standards. A decline in the use of these standards has been reported [1] as regionally developed standards are made available [2-5]. Mohan et al. (1990) have reported growth curves for North Indian babies from a referral hospital [1]. However, these reference standards have limitations as they failed to exclude mothers and babies with risk factors and the hospital predominantly served mothers with low socioeconomic status. Most of the above studies have not adhered to the World Health Organization (WHO) recommendation to base gestational age on number of completed weeks. The latest report on birth weight standards for South Indian babies was published in 1996, which is nearly one and a half decades old and may no longer be pertinent to infants born in more recent years [2]. The country has gone through economic revolution in the last two decades, which has influenced all sections of society. Therefore, the birth weight of the new born babies is expected to increase. Kramer et al. have reported the validity of the calculation of gestational age from the last menstrual period and therefore the credibility of the birth weight standards which were published earlier [3-6]. The gestational age from 24 weeks to 29 weeks are expected to be small in numbers and therefore likely to have a skewed distribution in birth weight. Some studies have failed to smooth the standard curves using appropriate distribution such as log normal or Box – Cox t distribution with cubic spline smoothing [7,8]. In this study we have overcome the above mentioned limitations by studying children born from 1996 to 2010 in a referral hospital which has a unique medical records system, with appropriate inclusion criteria, foetal sex and mother’s gravidity and gestation specific growth standards for Indian children.

## Methods

The Christian Medical College and Hospital is a referral hospital in South India, which caters to 47,110 outpatients and 15,662 inpatients per year. The department of Obstetrics and Gynaecology on an average delivered 20 babies per day in 1996 and 40 babies per day in 2010. The data was obtained from the Labour room register which was maintained by the nursing personnel and supervised by the Head of Obstetrics department. This contains all the information about women who delivered in this institution. Permission was obtained from all the Obstetric departmental Unit Heads. This study was approved by the ethics committee of [IRB Min. No. 7109 dated 10.03.2010] Christian Medical College.

### Sampling

The twelve months of the year were divided in to 4 quadrants. In the year 1996, all deliveries which took place in January were considered to represent the first quadrant. In the second quadrant, all deliveries in the month of April were considered. All deliveries in the months of July and October were considered to represent the deliveries in the third and fourth quadrants. In the second year of the study (1997) all deliveries which took place in February, May, August and November were taken to represent the first, second, third and fourth quarter of the year. In the third year of study (1998), all deliveries which took place in March, June, September and December were taken to represent the first, second, third and fourth quarter of the year. The above cycle was repeated for the next 3 years and continued until the year 2010 [9].

### Inclusion and exclusion criteria

Mothers aged 20 to 39 years and deliveries of gestational age between 24 weeks to 42 weeks were considered. The mothers, who had hypertensive disorders, gestational diabetes, diabetes mellitus, heart disease, and twin pregnancies, were excluded from the analyses. The gestational age specific birth weights which were above +3SD or below –3SD values were excluded from the analyses. The maternal height was regrouped into tertiles with categories as < 151 cm, 151 – 158 cm and > 158 cm based on the maternal height distribution of the mothers. Linear regression was employed to get the estimate of birthweight in relation to the maternal height. It showed a significant increase in birthweight of 135 grams. These 135 grams were added to the birthweight for shorter women and for taller women 135 grams were subtracted. Women with normal height (151–158 cm) did not have any adjustments in birthweight. These corrections yielded us the birthweights adjusted for maternal height.

The modified birthweights were adjusted with gestational ages to produce the birthweight centiles for each of the four groups in the term as well as the preterm groups. (Male & Female first born, Male & Female later born).

### Distribution and smoothing

The birth weights adjusted for maternal height for babies born from gestational age 24 weeks to 30 weeks had skewed distribution to the right side. However, as the number of deliveries increased in the subsequent gestational weeks, the birth weight distribution followed normal distribution. In the modelling we have assumed Box-Cox t distribution in order to get over this skewness. Cubic Spline smoothing has been applied using the Generalized Additive Models for Location Scale and Shape (GAMLSS) method using R software (Stasinopoulos and Rigby 2007) [10]. The GAMLSS models are semi parametric regression type models. However, the response variable needs to follow parametric distribution. The 3^rd^, 10^th^, 25^th^, 50^th^, 75^th^, 90^th^ and 97^th^ percentiles and mean and standard deviations were computed using R software. These centiles for birthweight adjusted for maternal height were done separately for firstborn males, females and later born males and females.

Data from the labour room register was entered using EPIINFO software. Completed weeks gestational age was considered for nomograms. The best estimate of gestation based on reliable menstrual history, early antenatal clinical examination and sonographic fetal biometry was used. Birth weights were measured to the nearest 50 g on a Braun electronic weighing scale within one hour of birth.

## Results

In total, there were 41,055 deliveries considered. Of these, complete data were available for 25,090 deliveries. When women with risk factors were excluded (mild and severe PIH, Chronic hypertension, GDM, Pregestational diabetics, cardiac disease, twins, teenaged primigravidas and mothers more than 40 years of age), 19,501 deliveries could be included for analyses. Most of the women were in the age group 25 – 29 years 41.7% (8133). Close to this is the 20 to 24 years age group which constituted 40.1% (7817). Mothers more than 30 years constituted only 18.2% (3551).

The maximum number of mothers who delivered belonged to the Hindu religion, and formed 82% (15933). Muslims formed 11.1% (2164), and Christians constituted 6.9% (1343).

Most (91.8%) of the mothers were housewives. Professionals were a minority, 4.3% (844), and mothers trained for skilled work were only 1.4% (241). When considering education of the mothers, 28.6% (4961) were graduates or more highly educated. A small percent was illiterate 5.1% (895). Those who had primary education formed 6.2% (1085) and those with secondary education 13.6% (2367). Those who had high school and higher secondary education constituted 8099 (46.5%) of the mothers.

When sub grouped into 3, based on the heights of the mothers, 24%, 48% and 28% of the mothers fell into the 3 groups ,whose height was <151 cm; 151-158 cm and >158 cm respectively.

There were 2379 firstborn and 17,092 later born. There were 1236 (6.3%) male babies in the first born group and the first born females were 1143 (5.9%) in number. Among the later born babies, male babies were 8739 (44.9%) in number and females 8353 (42.9%).

### Term and preterm first born babies

The male babies of term first born mothers were found to be 45 grams heavier than female babies. The mean birthweights were 2934 grams and 2889.5 grams respectively. Similarly, among the preterms, male babies weighed 152 grams more than the female babies. The mean birthweights were 1996 grams and 1844 grams respectively.

### Term and preterm later born babies

In the case of later born babies, term male babies weighed 116 grams more than the females. The mean birth weights were 3085 grams and 2969 grams respectively. When considering preterm babies among the later born, the males outweighed the female babies by 111 grams. The mean birthweights were 2089 grams and 1978 grams respectively.

**Figure 1:**
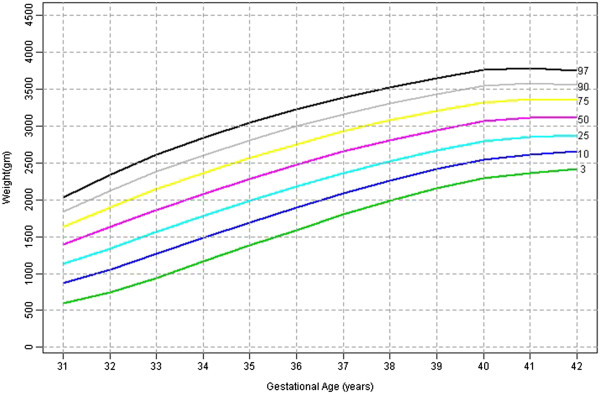
First born male babies smoothed centiles graph for Weight (gms) by gestational age (weeks).

**Figure 2:**
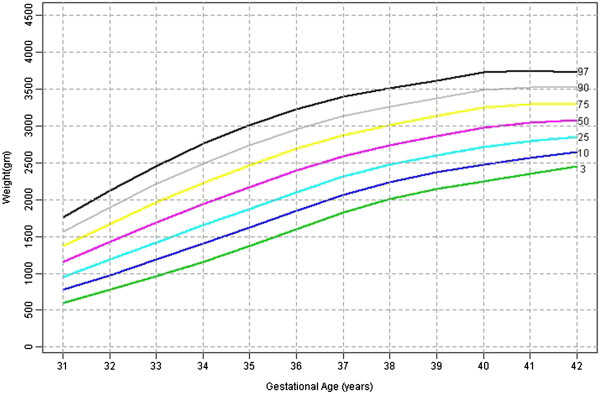
First born female babies smoothed centiles graph for weight (gms) by gestational age (weeks).

Table 1 shows the 3^rd^, 10^th^, 25^th^, 50^th^, 75^th^, 90^th^, 97^th^ percentile, mean and SD birth weights for male and female babies of primigravidae. Figures 1 and 2 shows the smoothed percentile curves for male and female babies of first born separately. The growth curves for various percentiles are smooth and increasing steadily as gestational age increases.

**Table 1:** Smoothed percentiles for birth weight (grams) of first born male and female babies

**First born male babies smoothed percentiles**											**First born female babies smoothed percentiles**									
**GA**	**N**	**C3**	**C10**	**C25**	**C50**	**C75**	**C90**	**C97**	**Mean**	**SD**	**N**	**C3**	**C10**	**C25**	**C50**	**C75**	**C90**	**C97**	**Mean**	**SD**
**31**	8	590	871	1131	1394	1637	1842	2035	1427	261	6	601	773	953	1159	1369	1562	1755	1146	310
**32**	13	744	1055	1340	1630	1899	2126	2340	1513	496	8	774	976	1187	1426	1670	1894	2119	1400	395
**33**	13	939	1262	1558	1861	2142	2381	2606	1820	529	10	961	1186	1421	1687	1958	2207	2456	1733	397
**34**	23	1164	1482	1777	2079	2362	2604	2833	2112	358	11	1157	1399	1649	1934	2223	2488	2754	2076	422
**35**	33	1379	1691	1981	2282	2566	2809	3039	2300	450	16	1366	1616	1874	2166	2464	2735	3007	1941	417
**36**	53	1591	1892	2176	2472	2752	2993	3222	2353	486	43	1598	1847	2104	2393	2688	2957	3225	2410	433
**37**	124	1806	2092	2365	2652	2925	3160	3385	2687	384	81	1821	2063	2311	2591	2875	3134	3392	2637	424
**38**	196	1982	2259	2524	2804	3072	3304	3525	2774	429	184	2004	2236	2474	2741	3012	3259	3505	2742	397
**39**	269	2152	2417	2673	2944	3204	3429	3645	2912	388	265	2142	2370	2604	2866	3132	3374	3615	2856	384
**40**	308	2287	2547	2798	3065	3321	3544	3758	3083	391	321	2250	2479	2714	2977	3244	3487	3729	2994	404
**41**	82	2366	2614	2855	3112	3359	3575	3781	3078	383	91	2353	2570	2792	3042	3294	3523	3751	3038	365
**42**	7	2419	2652	2877	3119	3352	3555	3751	2887	245	11	2448	2647	2851	3079	3310	3520	3728	3005	322

GA = Gestational Age (weeks), N = Number of babies in each group, C3 – C97 = Predicted Smoothed percentiles from GAMLSS model Mean & SD = Arithmetic Mean and standard deviation in each group.

**Figure 3:**
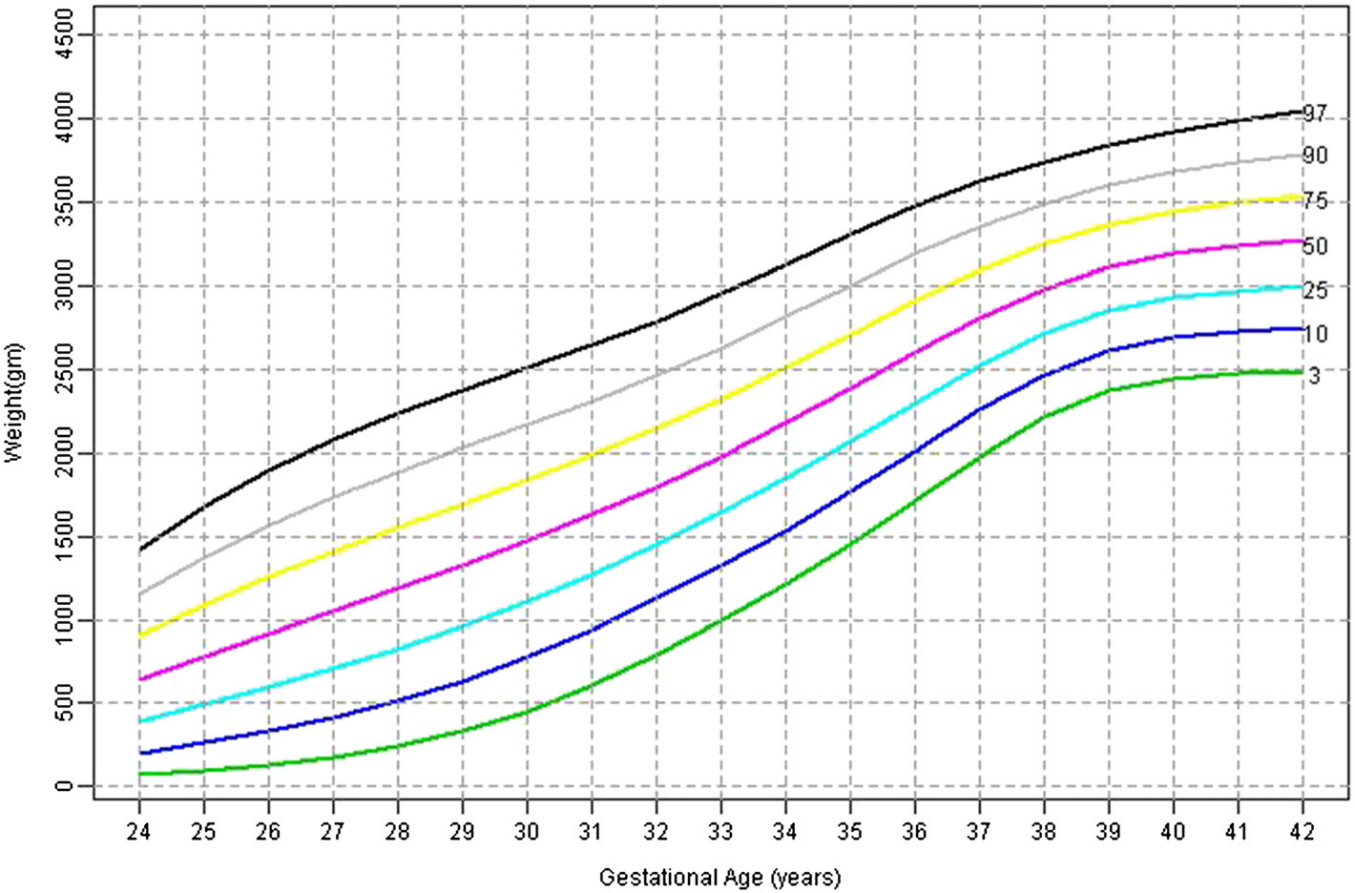
Later born male babies smoothed centiles graph for weight (gms) by gestational age (weeks).

**Figure 4:**
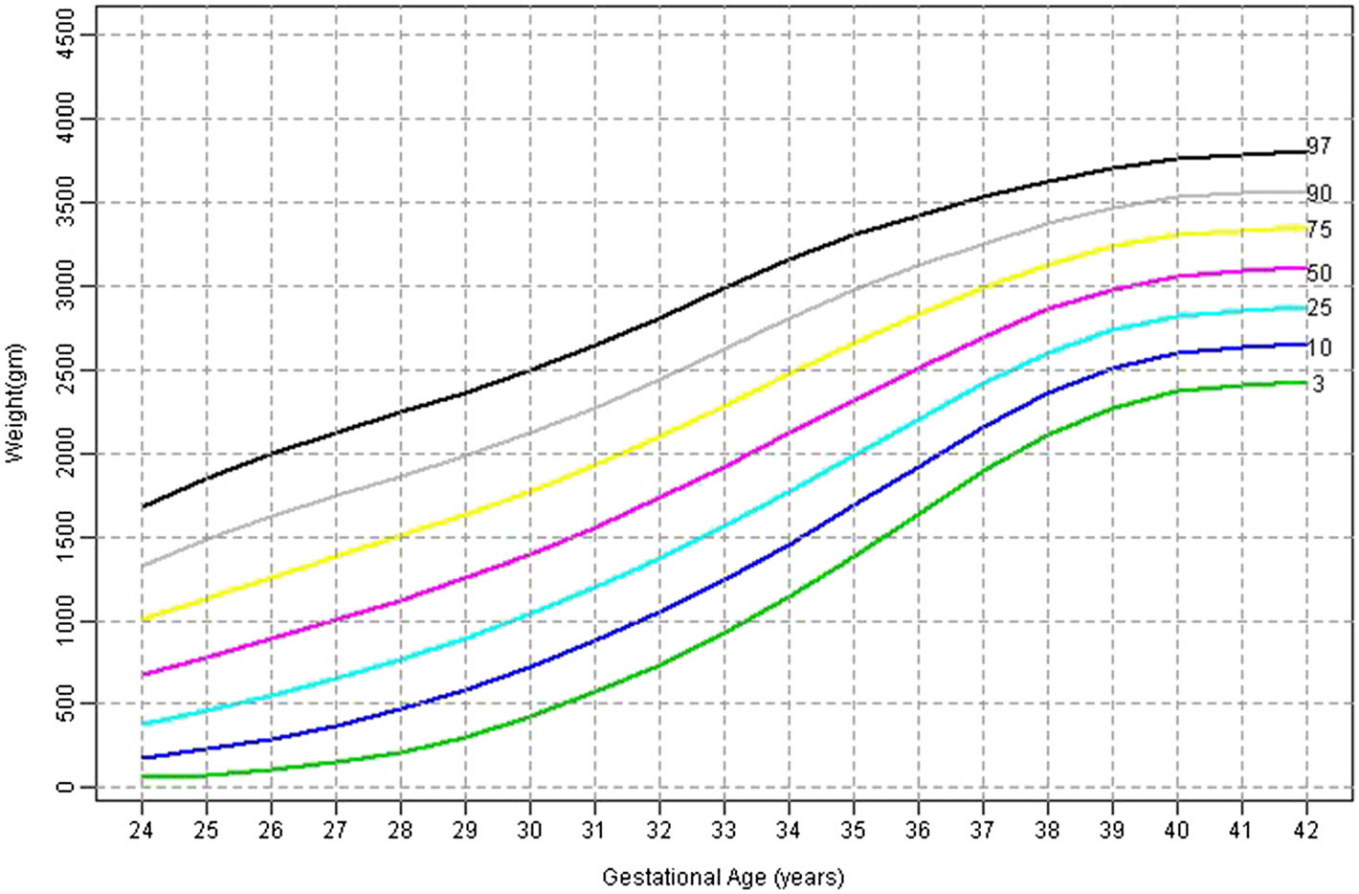
Later born female babies smoothed centiles graph for weight (gms) by gestational age (weeks).

Table 2 shows the 3^rd^, 10^th^, 25^th^, 50^th^, 75^th^, 90^th^, 97^th^ percentile, mean and SD birth weights for male and female babies of later born babies. Figures 3 and 4 shows the smoothed percentile curves for male and female babies of later born babies separately.

**Table 2:** Smoothed percentiles for birth weight (grams) of later born male and female babies

**Later born male babies smoothed percentiles**											**Later born female babies smoothed percentiles**									
**GA**	**N**	**C3**	**C10**	**C25**	**C50**	**C75**	**C90**	**C97**	**Mean**	**SD**	**N**	**C3**	**C10**	**C25**	**C50**	**C75**	**C90**	**C97**	**Mean**	**SD**
**24**	11	73	199	392	645	916	1172	1440	663	102	5	55	176	383	679	1018	1353	1714	792	133
**25**	9	99	259	491	785	1096	1388	1692	1099	645	8	75	226	464	787	1149	1503	1882	1136	833
**26**	14	131	328	594	920	1260	1578	1907	914	282	9	104	287	552	895	1270	1634	2021	893	490
**27**	16	175	409	704	1053	1412	1745	2090	1408	909	13	147	365	652	1007	1388	1753	2142	1217	836
**28**	22	237	508	824	1185	1552	1892	2242	1071	316	18	211	463	766	1126	1506	1869	2253	1208	500
**29**	14	327	629	958	1324	1691	2031	2381	1141	416	24	302	582	894	1256	1633	1991	2369	1195	480
**30**	45	450	775	1107	1470	1834	2168	2513	1595	721	41	421	720	1038	1399	1772	2126	2498	1470	695
**31**	39	610	943	1271	1626	1980	2306	2642	1567	387	51	567	878	1197	1557	1926	2275	2642	1479	451
**32**	60	795	1127	1449	1795	2140	2457	2785	1820	496	51	735	1052	1373	1731	2099	2444	2807	1621	469
**33**	97	997	1324	1639	1978	2316	2627	2947	1928	461	60	924	1244	1564	1920	2284	2626	2984	1956	578
**34**	104	1214	1536	1844	2176	2507	2812	3126	2137	556	116	1140	1455	1769	2117	2472	2804	3152	2116	570
**35**	187	1453	1765	2064	2386	2707	3002	3307	2314	479	160	1380	1684	1984	2316	2653	2968	3298	2307	519
**36**	323	1712	2008	2293	2600	2906	3188	3479	2552	506	232	1637	1922	2202	2511	2824	3117	3422	2468	506
**37**	745	1977	2253	2518	2805	3090	3353	3625	2808	453	599	1891	2154	2412	2696	2983	3251	3530	2692	433
**38**	1518	2210	2466	2713	2979	3244	3489	3741	2983	395	1399	2111	2355	2595	2857	3122	3369	3626	2867	396
**39**	2145	2369	2615	2852	3108	3363	3598	3841	3119	382	2164	2273	2505	2732	2980	3231	3465	3707	2981	377
**40**	2140	2445	2692	2930	3187	3443	3680	3924	3187	390	2160	2369	2595	2816	3058	3302	3529	3765	3078	366
**41**	475	2471	2725	2969	3233	3496	3739	3990	3205	421	507	2409	2632	2850	3090	3331	3555	3788	3040	370
**42**	53	2480	2742	2994	3266	3537	3788	4046	3080	391	57	2429	2651	2869	3106	3346	3569	3801	3019	404

GA = Gestational Age (weeks), N = Number of babies in each group, C3 – C97 = Predicted Smoothed percentiles from GAMLSS model Mean & SD = Arithmetic Mean and standard deviation in each group.

Table 3 presents the agreement between the model (adjusted) and actual data (unadjusted) percentiles for first born and later born male and female babies.

**Table 3:** Agreement between unadjusted and adjusted centiles

		**Adjusted centiles**				
		**< 10th**	**10 – 90th**	**> 90th**	**Weighted kappa**	**P value**
**First born male babies: (N = 1217)**						
**Unadjusted centiles**	**< 10th**	105	8	0		
	**10 – 90th**	20	943	18	0.883	< 0.001
	**> 90th**	0	6	117		
**Later born male babies: (N = 8739)**						
**Unadjusted centiles**	**< 10th**	810	43	0		
	**10 – 90th**	102	6791	101	0.917	< 0.001
	**> 90th**	0	25	867		
**First born female babies: (N = 1121)**						
**Unadjusted centiles**	**< 10th**	98	8	0		
	**10 – 90th**	12	874	16	0.897	< 0.001
	**> 90th**	0	6	107		
**Later born female babies: (N = 8353)**						
**Unadjusted centiles**	**< 10th**	787	10	0		
	**10 – 90th**	89	6551	65	0.934	< 0.001
	**> 90th**	0	37	814		

### Agreement of centiles for male & female babies among the first born babies

When considering first born male babies, there is significant agreement between adjusted and unadjusted percentile classification (k=.883, p<.001). However, 7% of the male babies in <10^th^ percentile category was misclassified as 10–90^th^ percentile category. Nearly 2% of the babies from 10–90^th^ percentile category was misclassified as <10^th^ and >90^th^ percentile. Nearly 5% of the babies >90^th^ percentile category was misclassified as 10–90^th^ percentile. When considering female babies, there is a significant substantial agreement between the adjusted and unadjusted classifications (k=.897, p<.001). The misclassifications to the next category percentiles rates were the same as in the male babies.

### Agreement of centiles for male & female babies within the later born group

There is a significant agreement between adjusted and unadjusted percentile classification (k=.917, p<.01) in later born male babies. However, 5% of the male babies in < 10^th^ percentile category was misclassified as 10–90^th^ percentile category and 1.5% from 10–90^th^ percentile category was misclassified as <10^th^ and >90^th^ percentile category. 2.8% of the >90^th^ percentile category was misclassified as 10–90^th^ percentile category. Similarly, among the later born female babies, there is a significant agreement between adjusted and unadjusted percentile classification (k=.934, p<.01). The misclassifications to the next category percentiles rates were nearly the same as among the male babies.

The mean (sd) of birthweights of the year 1996 was 2846 g (562) as compared to year 2010 (15 years later) which was 2907 (571), there was only a difference of 61 grams in the mean birthweights over one and half decades.

## Discussion

This is the biggest study ever from India, dealing with nearly 20,000 deliveries, including normal mothers with no antenatal risk factors from the same hospital covering 15 years. In addition to this, a major advantage is the reliance on early ultrasound-based estimates of gestational ages and appropriate statistical modelling using Box-Cox t distribution to get over the skewness of birth weight distribution and cubic spline smoothing. Therefore, we have established standards for first born and later born mothers for male and female babies separately. Some of the earlier standards have not done these adjustments [1,11] and some of the standards are very old [12,13]. The absence of downturn trend in the curves in the post term period is similar to curves reported [14] and are consistent with evidence based on early ultrasound-based gestational ages [15,16].

This study also compared the unadjusted centiles to adjusted and smoothed centiles. With male babies of first born, 7% of the adjusted <10^th^ percentile was misclassified as 10–90^th^ percentile and nearly each 2% of 10–90^th^ category was misclassified as <10^th^ or >90^th^ percentile category. A similar trend was obtained in female babies of first born and male and female babies of later born babies. Though there is very good agreement in general the highest misclassification rate was nearly 7.5%. Therefore the use of unadjusted percentiles may lead to unnecessary intervention and anxiety for the parents of babies whose weight fall in the range of lower and upper centiles according to adjusted centiles.

The limitation of the study is that the observations are cross-sectional. That is, birth weights of different babies were observed at different gestational ages at delivery. Ideally this has to be longitudinal in nature, that is, the same number of pregnancies and their birth weights have to be observed. Anthropometric measurements during gestation are feasible only using ultra-sound. However, the ultra-sound measurements have not been proved to be valid and reliable [17,18]. Temporal trends toward increasing maternal weight, weight gain during pregnancy due to various socio economic changes that have been taking place in the country in the last one and half decade needs to be studied.

With economic reforms, one expects improvement in birthweights. The mean (sd) of birthweights of the year 1996 was 2846 g (562) as compared to year 2010 (15 years later) which was 2907 (571), there was a difference of only 61 grams in the mean birthweights over one and half decades.

## Conclusion

New standards for birth weights of Indian newborns, both term and preterm have been established. In addition, birth weight standards based on the sex of the baby and maternal parity have also been brought out. These new standards will allow interventions to be based on Indian standards.

## Consent

Written informed consent was obtained from the patient for publication of this report.

## Competing interests

The authors declare that they have no competing interests.

## Authors’ contributions

All authors (VS, LJ, TS, AR, JM, RJ) contributed to the design of the study and interpretation of data. (VS, LJ, TS) performed the data analysis. (LJ, RJ) drafted the manuscript. All authors (VS, LJ, TS, AR, JM, RJ) critically revised the manuscript and have approved the final version.

## Publishing datasets

The dataset will not be available for publishing online since further research has been planned.

## Pre-publication history

The pre-publication history for this paper can be accessed here:

http://www.biomedcentral.com/1471-2393/13/38/prepub
